# Potentially Preventable Deaths by Intensive Care Medicine in Mongolian Hospitals

**DOI:** 10.1155/2016/8624035

**Published:** 2016-10-04

**Authors:** Naranpurev Mendsaikhan, Tsolmon Begzjav, Ganbold Lundeg, Martin W. Dünser

**Affiliations:** ^1^Intensive Care Department, Intermed Hospital, Ulaanbaatar, Mongolia; ^2^Division of Emergency Medicine and Anesthesia, Health Sciences University of Mongolia, Ulaanbaatar, Mongolia; ^3^Department of Critical Care, University College of London Hospital, London NW1 2BU, UK

## Abstract

*Purpose*. To evaluate the portion of hospitalized patients dying without prior intensive care unit (ICU) admission and assess whether death could have been prevented by intensive care.* Methods*. In this prospective, observational, multicenter study, data of adults dying in and outside the ICU in 5 tertiary and 14 secondary hospitals were collected during six months. A group of experts categorized patients dying without prior ICU admission as whether their death was potentially preventable or not.* Results*. 617 patients died (72.9% in and 27.1% outside the ICU) during the observation period. In 54/113 patients (32.3%) dying in the hospital without prior ICU admission, death was considered potentially preventable. The highest number of these deaths was seen in patients aged 16–30 years and those who suffered from an infection (83.3%), underwent surgery (58.3%), or sustained trauma (52%). Potentially preventable deaths resulted in a total number of 1,078 years of life lost and 709 productive years of life lost.* Conclusions*. Twenty-seven percent of adults dying in Mongolian secondary and tertiary level hospitals do so without prior ICU admission. One-third, mostly young patients suffering from acute reversible conditions, may have potentially been saved by intensive care medicine.

## 1. Introduction

Mongolia is a Central Asian landlocked country which is home to approximately three million people [1]. Intensive care medicine in Mongolia is an underdeveloped medical specialty [2, 3]. In a recent nationwide survey, our study group observed that the adult and pediatric ICU bed capacity in Mongolia [11.7 intensive care unit (ICU) beds per 100,000 inhabitants] compares favorably with other low- and middle-income countries. However, the fact that only half of the ICU beds were equipped with mechanical ventilators indicates important resource restrictions in Mongolian ICUs (unpublished results). Critical illness in Mongolia affects younger patients compared to high-income countries. ICU admission diagnoses are similar with a particularly high incidence of stroke and liver failure. ICU mortality is approximately 25% with most deaths caused by stroke, liver failure, and traumatic brain injury (unpublished results). It is currently unknown whether critically ill patients in Mongolia are routinely referred to the ICU service or remain in non-ICU wards for treatment.

In this multicenter study, we evaluated the portion of patients who died in Mongolian hospitals without prior admission to the ICU and, based on these data, estimated the number of deaths which could have potentially been prevented by intensive care.

## 2. Materials and Methods

This analysis was designed as a prospective, observational, multicenter study. It was conducted in five tertiary and five secondary level hospitals in the capital city of Mongolia as well as in nine secondary level provincial hospitals. All study-related data were collected during a six-month period from September 1, 2014, until February 28, 2015. The study protocol was approved by the Ethics Committee of the Mongolian National University of Medical Sciences. As only anonymous data were collected and no therapeutic changes were performed, written informed consent was waived by the committee.

### 2.1. Study Patients

All patients who were hospitalized and died in one of the study hospitals during the observation period were eligible for study inclusion. Patients <16 years were excluded.

### 2.2. Data Collection

Local physician site investigators collected the following data in all study patients: age, gender, comorbid conditions, reason for hospital admission, length of hospital stay, treatment costs (in Mongolian Togrog and US dollars by exchange rate December 2015; not including staff or ICU maintenance costs), and cause of death. Autopsy results were documented whenever a postmortem analysis had been performed. The admission diagnoses were categorized into six diagnostic categories [neurological, medical, trauma, surgical (nontrauma), infection, and miscellaneous]. Based on the results of a previous prospective observational study, the predicted absolute risk of death was calculated for each diagnostic category. We chose this approach as calculation of the individual risk of death using validated intensive care scores (e.g., SAPS, APACHE) depends on laboratory results which are not routinely available in the majority of Mongolian ICUs [2, 3].

In patients admitted to the ICU prior to death, the reason for ICU admission, administration of oxygen, and/or vasopressors and the length of mechanical ventilation and ICU stay were documented.

### 2.3. Evaluation of the Potential Preventability of Death

Medical charts of patients dying without prior ICU admission were reviewed by a panel of four intensivists with experience working in tertiary and secondary level hospitals both in the capital city and in various provinces of Mongolia. This group of experts determined whether the death of patients could have been prevented by ICU admission and subsequent intensive care. Disagreement was resolved by discussion and final consensus. In case no consensus could be reached, death was assumed to have been unpreventable. To calculate the number of productive years of life lost and the number of years of life lost that could have been saved, the number of potentially preventable deaths was adjusted for the predicted risk of death. This predicted risk of death was calculated for each diagnostic category and assumed that the patient received intensive care. The number of productive years of life lost was then calculated as the difference between the average age at retirement in Mongolia (male, 60 years; female, 55 years) and the age at death [4]. The number of years of life lost was calculated as the average life expectancy at birth in Mongolia in 2014 (male, 64.7 years; female, 73.5 years [5]) and the age at death [4].

### 2.4. Statistical Analysis

The SPSS software package was used for all statistical analyses (SPSS Inc.; SPSS IBM, Chicago, Illinois, United States of America). Descriptive methods were used to present data with categorical variables given as absolute numbers and percentage and continuous variables as median values with interquartile ranges. Comparisons between patients dying with or without prior ICU admission and between patients whose death was considered potentially preventable or not were made using the Mann-Whitney* U*, Fisher's Exact, or* Chi* ^2^ test, as appropriate. *p* values <0.05 were considered to indicate statistical significance.

## 3. Results

During the observation period, 118,411 patients were admitted to the study hospitals (median per study site, 5,345; IQR, 3,823–8,613) of whom 617 patients died resulting in an overall hospital mortality of 0.5%. Five-thousand-four-hundred-thirty-three of all hospitalized patients were admitted to the ICU (median, 2.1%; IQR, 1.1–2.9%) resulting in an overall ICU mortality of 8.3%. Of all patients dying in the hospital, hundred-sixty-seven patients did so without prior ICU admission (27.1%). These patients were more often treated in secondary level hospitals and less frequently in hospitals where an intensivist was available, differed in their comorbidities, admission categories, and causes of death, with a lower predicted risk of death, and had a longer hospital length of stay than patients dying in the ICU (Table 1). Autopsies were performed less frequently in patients dying without prior ICU admission than in those who died in the ICU. Admission reasons and interventions administered to patients who died in the ICU are displayed in Table 2.

**Figure 1:**
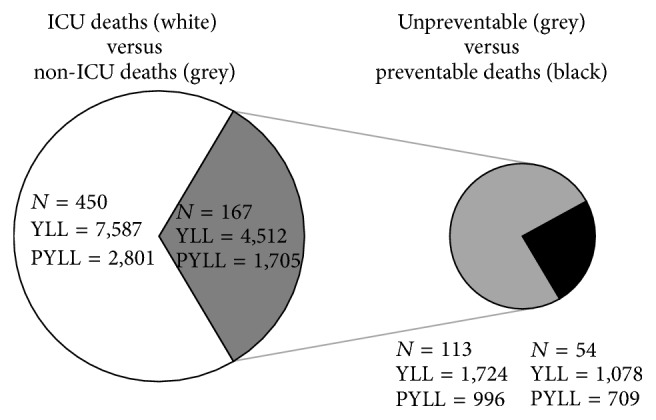
Portions of ICU deaths, non-ICU deaths, and unpreventable and preventable death cases in the study population, as well as absolute numbers of (productive) years of life lost. ICU, intensive care unit; YLL, years of life lost; PYLL, productive years of life lost.

**Figure 2:**
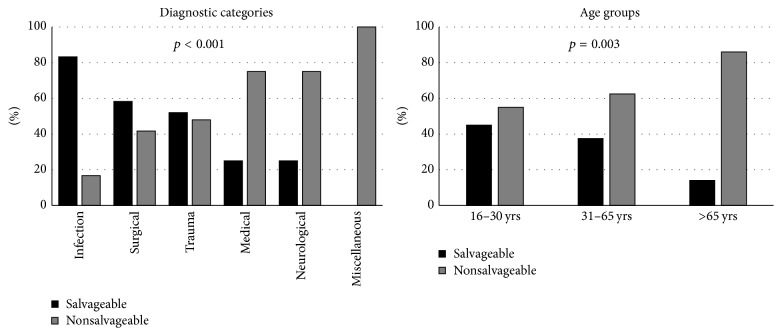
Ratio between potentially preventable (black bars) and unpreventable (grey bars) deaths by diagnostic categories and age groups.

In fifty-four of 167 patients dying without prior ICU admission (32.3%) death was considered potentially preventable by intensive care (Figure 1). Study patients whose death was potentially preventable were less often treated in hospitals where an intensivist was available, were younger and more often male, differed in their comorbid conditions and diagnostic categories, and had a longer length of hospital stay and a higher predicted risk of death than patients in whom death was considered unpreventable (Table 3). Autopsies were more often performed in patients whose death was considered potentially preventable. Diagnostic categories and age groups differed between patients whose death was considered potentially preventable or unpreventable (Figure 2). The highest numbers of potentially preventable death cases were observed in the age groups 16–30 and 31–65 years as well as in the diagnostic categories of infection, surgery, and trauma.

## 4. Discussion

The most striking finding of this prospective, observational multicenter study was that twenty-seven percent of all study patients who died in the participating Mongolian secondary and tertiary level hospitals did so without prior ICU admission, even though their death may have been preventable by intensive care. The fact that the majority of these patients were young adults suffering from reversible acute conditions such as infection, surgical disease, or trauma underlines the importance of this observation. The associated socioeconomic impact of such premature deaths is substantial as indicated by the number of (productive) years of life lost.

Given the ethical impossibility to perform a randomized controlled trial using ICU admission as an intervention in critically ill hospitalized patients, we chose to retrospectively classify death cases occurring in the study hospitals outside of the ICU as either potentially preventable or unpreventable. This classification was done by an expert panel of experienced Mongolian intensivists. Although it is well conceivable that this panel may have wrongly judged the reversibility of the acute disease condition of some study patients, the experts were rather conservative in their consensual decisions to avoid overestimation of the potential preventability of death cases by intensive care. A lower median age, less comorbid conditions, more reversible pathologies as diagnostic categories, and a longer hospital stay in patients whose death was considered potentially preventable compared to those in whom death was rated as unpreventable suggest that the expert panel's decision process was clinically reasonable. On the other hand, there are arguments that the number of preventable deaths in the study hospitals during the observation period may have been underestimated by our analysis. For example, our study did not assess the number of patients who died prior to hospital admission or in referring primary care hospitals that run no ICU service and usually transfer critically ill patients to the study hospitals unless impeded by weather or other logistical challenges. Finally, we cannot exclude that some death cases occurring in the study hospitals during the observation period were not reported.

We did not document reasons for not admitting critically ill hospitalized patients to the ICU. Therefore, it is unclear whether transfer to the ICU was not performed due to logistical reasons, inappropriate triage, or other reasons (e.g., financial barriers). The result, however, that the presence of an intensivist in the study hospital was related to a lower rate of potentially preventable deaths could point at the possibility that inappropriate triage of patients on non-ICU wards has played a role.

It is important to emphasize that the expert panel based its decision on the potential preventability of deaths by intensive care assuming ICU facilities as contemporarily encountered in Mongolia [2, 3]. In previous analyses, our study group reported that intensive care medicine in Mongolia faces serious challenges such as a lack of resources [2, 3]. Accordingly, interventions known to save lives in critically ill patients, such as mechanical ventilation [6], vasopressor therapy [7], or renal replacement therapy [8], were set in only a comparatively small amount of study patients dying in the ICU. For example, the observation that only 66% of study patients dying in the ICU were mechanically ventilated may reflect the reported lack of mechanical ventilators in Mongolian ICUs. These results could further be one reason for the striking finding that total costs of care did not differ between patients dying with or without prior ICU admission.

Twenty-seven percent of study patients died in the hospital without prior ICU admission. While this number compares well to publications from North America [9] and Brazil [10], a much lower rate was found in a rural Sub-Saharan African setting. Only ~10% of patients dying in an Ugandan hospital did so after ICU admission. The number of ICU beds per 100,000 inhabitants may delicately influence this ratio. Given the comparatively high number of available adult ICU beds per 100,000 inhabitants (*n* = 8.1 [11]) in Mongolia compared to other countries (Uganda, *n* = 0.1 [12]; Spain, *n* = 8.2 [13]; United States, *n* = 20 [13]), it is unlikely that the lack of ICU beds has been the single factor why young patients dying from a potentially reversible acute disease had not been referred to an ICU in our analysis.

## 5. Conclusions

This prospective, observational, multicenter study suggests that twenty-seven percent of adult patients dying in Mongolian secondary and tertiary level hospitals do so without prior ICU admission. An expert intensivist panel considered that one-third of these patients, mostly young patients suffering from acute reversible conditions, may have potentially been saved by intensive care.

## Figures and Tables

**Table 1:** Demographic and clinical data of hospitalized adults dying in and outside of the ICU.

Parameter	ICU deaths	Non-ICU deaths	*p* value
*N*	450	167	
Level of hospital (*n*/%)			0.04^*∗*^
* Level II*	163 (36.2)	76 (45.5)	
* Level III*	287 (63.8)	91 (54.5)	
Intensivist available (*n*/%)	407 (90.4)	135 (80.8)	0.002^*∗*^
Age (years)	52 (42–63)	53 (41–67)	0.53
Male gender (*n*/%)	289 (64.2)	98 (58.7)	0.22
Comorbid conditions (*n*/%)			0.04^*∗*^
* None*	130 (28.9)	35 (21)	
* Arterial hypertension*	115 (25.6)	45 (26.9)	
* Liver cirrhosis*	67 (14.9)	25 (15)	
* Congestive heart failure *	44 (9.8)	18 (10.8)	
* Diabetes mellitus*	29 (6.4)	11 (6.6)	
* Chronic renal insufficiency*	27 (6)	7 (4.2)	
* Cancer*	19 (4.2)	20 (12)	
* Chronic respiratory insufficiency*	17 (3.8)	6 (3.6)	
Diagnostic category (*n*/%)			<0.001^*∗*^
* Neurological*	142 (31.6)	52 (31.1)	
* Medical*	130 (28.9)	44 (26.3)	
* Trauma*	92 (20.4)	25 (15)	
* Surgical (nontrauma)*	38 (8.4)	12 (7.2)	
* Infection*	33 (7.3)	12 (7.2)	
* Miscellaneous*	15 (3.3)	22 (13.2)	
Hospital length of stay (days)	2 (1–6)	3 (1–8)	<0.001^*∗*^
Treatment costs			
* Tugrik (thousands)*	235 (88–321)	230 (80–279)	0.4
* US dollars (exchange rate December 15)*	117 (44–161)	115 (40–140)	0.4
Predicted risk of death by diagnostic category	0.29 (0.23–0.3)	0.28 (0.21–0.3)	0.03^*∗*^
Cause of death (*n*/%)			0.007^*∗*^
* Multiple organ failure*	225 (50.1)	67 (40.1)	
* Coma*	105 (23.3)	53 (31.7)	
* Shock*	99 (22)	31 (18.6)	
* Respiratory failure*	20 (4.5)	16 (9.6)	
Autopsy performed (*n*/%)	221 (49.1)	60 (35.9)	0.003^*∗*^

ICU, intensive care unit; ^*∗*^significant difference between hospitalized adults dying in and outside of the ICU.

Data are given as median values with interquartile range, if not otherwise indicated.

**Table 2:** Admission reasons and interventions set in study patients dying in the ICU.

*N*	450
Reasons for ICU admission (*n*/%)	
* Coma*	222 (49.4)
* Shock*	123 (27.4)
* Respiratory failure*	42 (9.4)
* Postoperative status*	35 (7.8)
* Multiple organ dysfunction syndrome*	18 (4)
* Cardiac arrest*	9 (2)
Oxygen administration (*n*/%)	151 (33.6)
Mechanical ventilation (*n*/%)	299 (66.4)
Length of mechanical ventilation (days)	1 (1–3)
Vasopressor therapy (*n*/%)	195 (43.3)
Type of vasopressor administered (*n*/%)	
* Epinephrine*	103 (22.9)
* Dopamine*	70 (15.6)
* Norepinephrine*	5 (1.1)
* Other*	16 (3.6)
Length of ICU stay (days)	1 (1–3)

ICU, intensive care unit.

Data are given as median values with interquartile range, if not otherwise indicated.

**Table 3:** Demographic and clinical data of study patients dying outside of the ICU.

Parameter	Potentially preventable patients	Unpreventable patients	*p* value
*N*	54	113	
Intensivist available (*n*/%)	38 (70.4)	97 (85.8)	0.02^*∗*^
Age (years)	48.5 (36.3–62)	56 (41.5–72)	0.006^*∗*^
Male gender (*n*/%)	40 (74.1)	58 (51.3)	0.007^*∗*^
Comorbid conditions (*n*/%)			<0.001^*∗*^
* None*	21 (38.9)	14 (12.4)	
* Arterial hypertension*	18 (33.3)	27 (23.9)	
* Liver cirrhosis*	2 (3.7)	23 (20.4)	
* Congestive heart failure*	1 (1.9)	17 (15)	
* Diabetes mellitus*	4 (7.4)	7 (6.2)	
* Chronic renal insufficiency*	2 (3.7)	5 (4.4)	
* Cancer*	3 (5.6)	17 (15)	
* Chronic respiratory insufficiency*	3 (5.6)	3 (2.7)	
Diagnostic category (*n*/%)			<0.001^*∗*^
* Neurological*	13 (24.1)	39 (34.5)	
* Medical*	11 (20.4)	33 (29.2)	
* Trauma*	13 (24.1)	12 (10.6)	
* Surgical (nontrauma)*	7 (13)	5 (4.4)	
* Infection*	10 (18.5)	2 (1.8)	
* Miscellaneous*	0	22 (19.5)	
Hospital length of stay (days)	5 (3–9)	3 (1–7)	0.002^*∗*^
Treatment costs			
* Tugrik (thousands)*	230 (89–620)	230 (61–273)	0.17
* US dollars (exchange rate December 15)*	115 (45–310)	115 (31–137)	0.17
Predicted risk of death by diagnostic category	0.29 (0.21–0.37)	0.28 (0.2–0.29)	0.047^*∗*^
Years of life lost	21 (9–29)	13 (0–25)	0.19
Productive years of life lost	10 (0–23)	1 (0–18)	0.16
Autopsy performed (*n*/%)	27 (50)	33 (29.2)	0.02^*∗*^

ICU, intensive care unit; ^*∗*^significant difference between patients whose death was considered potentially preventable and those whose death was considered unpreventable.

Data are given as median values with interquartile range, if not otherwise indicated.
